# Electrocatalytic Performance of MnMoO_4_-rGO Nano-Electrocatalyst for Methanol and Ethanol Oxidation

**DOI:** 10.3390/molecules28124613

**Published:** 2023-06-07

**Authors:** Parisa Salarizadeh, Sadegh Azizi, Hossein Beydaghi, Ahmad Bagheri, Mohammad Bagher Askari

**Affiliations:** 1 High-Temperature Fuel Cell Research Department, Vali-e-Asr University of Rafsanjan, Rafsanjan P.O. Box 7718897111, Iran; 2 Department of Physics, Faculty of Science, University of Guilan, Rasht P.O. Box 41335-1914, Iran; 3 BeDimensional SpA, Lungotorrente Secca 30R, 16163 Genoa, Italy; 4 Graphene Labs, Istituto Italiano di Tecnologia, Via Morego 30, 16163 Genoa, Italy; 5 Department of Semiconductor, Institute of Science and High Technology and Environmental Sciences, Graduate University of Advanced Technology, Kerman P.O. Box 76318-85356, Iran

**Keywords:** MnMoO_4_, rGO, methanol oxidation reaction, ethanol oxidation reaction

## Abstract

Today, finding low-cost electro-catalysts for methanol and ethanol oxidation with high performance and stability is one of the new research topics. A nanocatalyst based on metal oxides in the form of MnMoO_4_ was synthesized by a hydrothermal method for methanol (MOR) and ethanol (EOR) oxidation reactions. Adding reduced graphene oxide (rGO) to the catalyst structure improved the electrocatalytic activity of MnMoO_4_ for the oxidation processes. The crystal structure and morphology of the MnMoO_4_ and MnMoO_4_-rGO nanocatalysts were investigated by physical analyses such as scanning electron microscopy and X-ray diffraction. Their abilities for MOR and EOR processes in an alkaline medium were evaluated by performing electrochemical tests such as cyclic voltammetry, chronoamperometry, and electrochemical impedance spectroscopy. MnMoO_4_-rGO showed oxidation current densities of 60.59 and 25.39 mA/cm^2^ and peak potentials of 0.62 and 0.67 V in MOR and EOR processes (at a scan rate of 40 mV/s), respectively. Moreover, stabilities of 91.7% in MOR and 88.6% in EOR processes were obtained from the chronoamperometry analysis within 6 h. All these features make MnMoO_4_-rGO a promising electrochemical catalyst for the oxidation of alcohols.

## 1. Introduction

Excessive consumption and indiscriminate extraction of fossil fuel resources for the supply of energy will see human societies facing an energy crisis in the coming years. In addition, excessive consumption of fossil fuels has a direct effect on the spread of greenhouse gases and the creation of various respiratory diseases. Every year, in many international communities, solutions are presented to prevent damages caused by energy shortages in the coming years. One of the best and most accessible ways to improve energy emergencies is to use renewable and clean fuel sources. There are sustainable resources such as sunlight, wind, earth’s heat, tides, etc., which different countries use according to their geographical location. One of the essential advantages of renewable resources is their availability for all countries and that under political or economic problems, these resources are still available. One of the biggest concerns facing countries is use of technologies that can convert energy with the highest efficiency. Getting to grips with the technology of storing and producing energy from clean fuels requires extensive knowledge, which many scientists are engaged in research in this field. The remarkable progress of recent years in the field of introducing high-tech devices in energy production is a confirmation of the efforts of scientists in this field. The introduction of solar cells with the highest efficiency, wind turbines in different dimensions, and tools for converting geothermal energy into electricity are among the most significant studies in the field of energy.

With the development of nanoscience and the great interest of researchers in this branch of science, a huge revolution has been created in various sciences. The application of nanomaterials and nanocomposites in sciences and industries such as health, food science, biology and biotechnology, military and telecommunication industries, air and space, etc., represents an example of the wide application of materials science. Undoubtedly, one of the most widely used fields in nanoscience is the application of these materials in the energy field. The applications of nanomaterials in devices such as supercapacitors, batteries, and types of fuel cells are examples of the wide application of nanoscience in the energy field.

There are different types of fuel cells, including proton-exchange membrane fuel cells (PEMFC), formic acid fuel cells (FFC), alcohol fuel cells (AFC), molten carbonate fuel cells (MCFC), microbial Fuel Cells (MFC), phosphoric acid fuel cell (PAFC), air–zinc fuel cell (Zn–Air FC), solid oxide fuel cell (SOFC) and Ceramic Fuel Cells Ltd. (ASX: CFU). Meanwhile, alcohol fuel cells are more popular than others due to their lower operating temperature and simple structure.

Alcoholic fuel cells have received a lot of attention in the world of science and technology due to their extraordinary advantages, such as high energy density [1], relatively small dimensions [2], easy transportation, environmental compatibility, etc. [3,4,5]. These devices produce electricity directly in an electrochemical reaction [6]. The fuels used in fuel cells can be all kinds of alcohol, such as methanol, ethanol, sugar alcohols, etc. Among these fuels, methanol and ethanol are of more interest due to their simple molecular structure and relatively easy production, availability, and low cost [2,7].

Alcoholic fuel cells consist of three main parts: anode, cathode, and polymer membrane [8]. The alcohol oxidation process takes place in the anode of alcohol fuel cells [9], and the cathode has the task of oxygen reduction [10]. There is a polymer membrane (generally Nafion) between the anode and the cathode, which performs the function of proton transfer [11]. The result of the operation of a such system is the production of electricity with byproducts such as water and heat [12]. Therefore, a clean and environmentally friendly system can undoubtedly be an excellent option to solve the problems of the energy crisis and overcome environmental pollution.

As mentioned, the oxidation process of alcohols such as methanol and ethanol occur in the anode of alcohol fuel cells. Catalysts that are generally known and more productive in this field are platinum-based materials. Other expensive and rare metals such as palladium, ruthenium, etc., can also oxidize alcoholic fuels in the best way [13,14]. Expense and poisoning of the surface of these catalysts are two very important obstacles in the industrialization of fuel cells. Hence, researchers are looking for alternative catalysts to the platinum family.

In many studies, attempts have been made to reduce the amount of platinum in the catalyst structure, and by hybridizing it with carbon substrates [15,16], metal oxides [17,18], metal sulfides [19], or conductive polymers [20], a goal that has been achieved to some extent.

In the field of catalyst science, metal oxides have shown excellent performance. Their wide use in sensing [21,22] or electrochemical oxidation and reduction processes [23,24,25,26] is the proof of the wide application of these materials. In the energy field, metal oxides in the structure of the electrodes of supercapacitors are of great interest [27,28].

Relatively easy synthesis, low cost, abundance, and availability, along with theoretical and simulation studies that confirm their excellent efficiency in energy storage in supercapacitors, are the most important reasons for the push toward metal oxide precursors [29]. Despite the wide application of metal oxides in various fields, so far, few studies have investigated their use in the oxidation of methanol and ethanol for use in the anode of alcohol fuel cells.

According to what was mentioned, much attention has been paid to the identification and preparation of abundant and cheap catalytic materials based on transition metal compounds such as transition metal sulfides and transition metal oxides, especially mixed metal oxides, which has drawn a lot of attention owing to their higher conductivity and active sites compared to commonly used single oxide compounds. For example, Li et al. prepared a two-dimensional Mn_2_O_3_@δ-MnO_2_ core-shell structure to investigate methanol oxidation. The interaction between metal oxides improves the catalytic activity, the chemical properties, and the electronic structure of the catalyst [30]. Jinxi et al. prepared ZnFe_2_O_4_-ZrO_2_ with enhanced electrochemical performance, due to high conductivity and quick electron transit [31]. Mu et al. investigated carbon nanotubes/reduced graphene oxide/MnMoO_4_ composites with excellent stability for supercapacitors, due to the synergistic effects from each component of the composite [32]. Moreover, in our previous works, we synthesized some binary transition metal oxides such as RuO_2_-MnCo_2_O_4_/rGO [29], MgCo_2_O_4_/rGO [33], and MnNi_2_O_4_/rGO [34], with good electrochemical performance owning to catalytic activity and synergic effect between the catalyst components. Unlike single metal oxides, particular bimetallic compounds have established identical promising performances and stabilities.

Moreover, in order to improve the performance of catalysts, it is necessary to improve their active surface area and electrical conductivity. Micro and nanostructured carbonaceous materials such as multiwalled carbon nanotubes, graphene, reduced graphene oxide, carbon dots, hollow carbons, etc., have been considered as a support for transition metal oxides/sulfides due to high electrical conductivity. Furthermore, using carbon supporting materials decreases the aggregation of active sites, provides high surface area. In addition to these beneficial features, carbon materials can be easily prepared with low cost. Thus, the use of various types of carbons is of great interest to researchers in the field of energy due to the abundance of this element in nature and the extraordinary physical and chemical properties of this material.

In this research, MnMoO_4_ and MnMoO_4_-rGO are hydrothermally synthesized in one step, and after performing the physical characterization, the capability of these catalysts in the methanol (MOR) and ethanol (EOR) oxidation reaction processes is investigated through detailed electrochemical tests. The results show the good potential of the introduced catalysts for methanol oxidation. The introduction of platinum-free catalysts with relatively good efficiency can open a new way in the field of energy production in fuel cells. 

## 2. Results and Discussion

As mentioned, MnMoO_4_ and MnMoO_4_-rGO were synthesized by the hydrothermal method. In this part, after examining the crystal structure of nanocatalysts by X-ray diffraction (XRD) analysis, the morphology of these catalysts is examined by providing SEM and TEM images. Catalyst performance in the methanol oxidation reaction (MOR) and ethanol oxidation reaction (EOR) process is evaluated by cyclic voltammetry (CV), electrochemical impedance spectroscopy (EIS), and linear sweep voltammetry (LSV) analyses. First, the behavior of the catalyst in the absence of methanol and ethanol in an alkaline environment is examined, and then the oxidation process of alcohols by adding methanol and ethanol to the potassium hydroxide (KOH) solution is evaluated. Finally, the stability of nanocatalysts as well as the effect of temperature on methanol and ethanol oxidation is investigated. The results of electrochemical analyses in this research show the positive effect of adding rGO to the catalyst structure. By increasing the electrochemical active surface area of the catalyst and improving its electrical conductivity, the rGO nanosheet improves the efficiency and performance of the catalyst and, thus, facilitates the oxidation processes of methanol and ethanol.

### 2.1. Characterization of Catalysts

**Figure 1:**
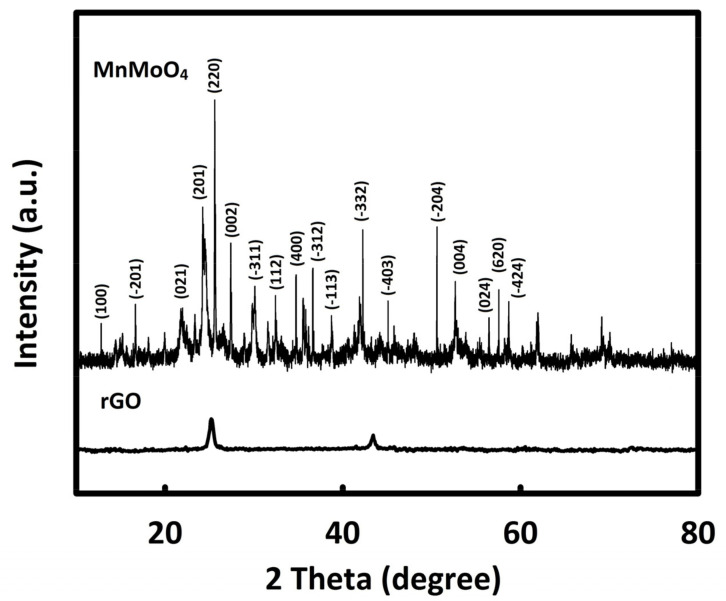
X-ray diffraction patterns of rGO and MnMoO_4_.

The crystal structure of MnMoO_4_ and rGO was evaluated by X-ray diffraction (XRD) analysis (Figure 1). The characteristic peaks of MnMoO_4_ can be seen at 13°, 18.5°, 22.5°, 24.6°, 25.7°, 26°, 31.2°, 34.1°, 35.6°, 38°, 39.1°, 42.7°, 45.4, 51.3°, 53.4°, 57.0°, 58.3°, and 59.3°, which are consistent with JCPDS card no: 01-72-0285 [35]. The average size of MnMoO_4_ crystals was estimated to be 28 nm using the Scherrer equation (D = Kλ/βcosθ) [36]. Furthermore, in the XRD pattern of reduced graphene oxide, two characteristic peaks can be seen at the diffraction angles of about 26° and 43°.

**Figure 2:**
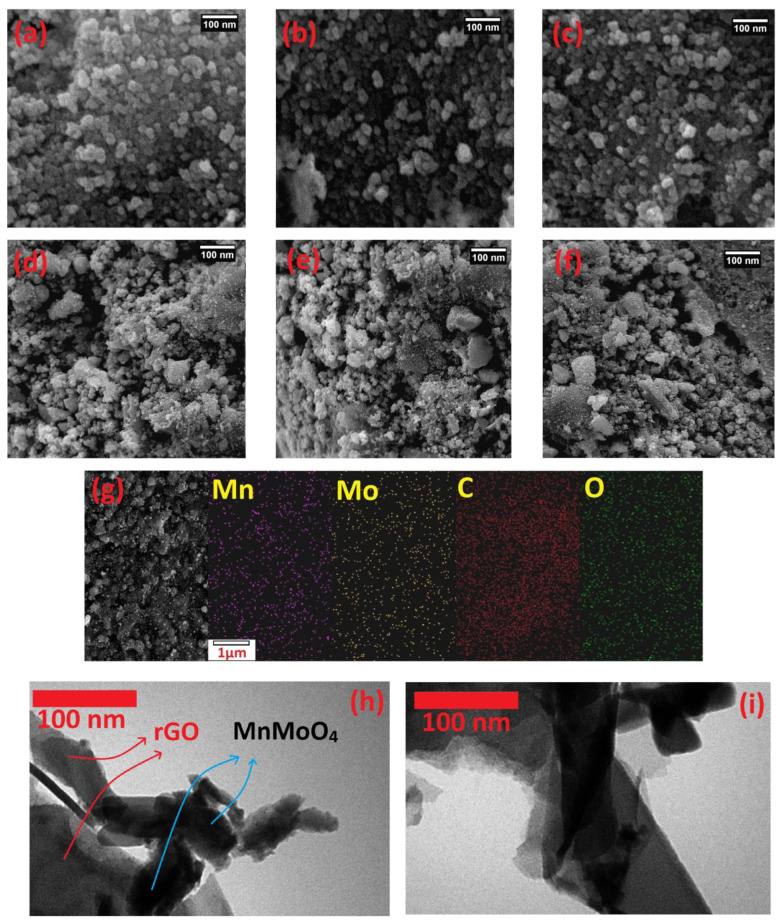
SEM images of MnMoO_4_ (**a**–**c**), MnMoO_4_-rGO (**d**–**f**), and EDS mapping of MnMoO_4_–rGO (**g**). TEM images of MnMoO_4_-rGO (**h**,**i**).

To investigate the morphology of MnMoO_4_ and MnMoO_4_-rGO, scanning electron microscope SEM and TEM images of the catalysts were prepared. Figure 2a–c belongs to the MnMoO_4_ catalyst in scales of 100 nm. The presence of porosity, which is a very important factor in facilitating electrochemical processes, is clearly evident in these images and in the structure of MnMoO_4_. The placement of MnMoO_4_ on the rGO surface is evident in the SEM images (Figure 2d–f). In these images, MnMoO_4_ almost uniformly covers the surface of rGO nanosheets. Figure 2g show energy dispersive X-ray spectroscopy (EDS) mapping images of MnMoO_4_–rGO nanocatalyst. From this figure, the presence of manganese, molybdenum, oxygen, and carbon elements in the catalyst structure is confirmed. Moreover, the uniform distribution of these elements on the rGO surface is seen.

Figure 2h,i is related to transmission electron microscope (TEM) images of synthesized MnMoO_4_–rGO. The rGO nanosheets with plate morphology is seen in these images. The transparency of these sheets indicates the very low thickness of the rGO sheets. Moreover, presence of MnMoO_4_ nanoparticles on the rGO surface can be seen.

**Figure 3:**
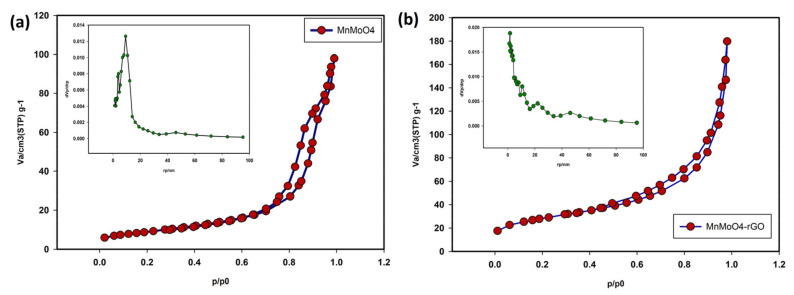
BET and BJH curves MnMoO_4_ (**a**), MnMoO_4_-rGO (**b**).

The specific surface area was investigated using the N_2_ adsorption–desorption. Figure 3a is related to the MnMoO_4_ sample, which, compared to the standard IUPAC isotherms, shows that the isotherm of the synthesized composite is type IV, and using the BET method, the effective surface area was also 57 m^2^/g. Moreover, using the BJH method (inset of Figure 3a), the average pore size for MnMoO_4_ was about 4.7 nm. BET and BJH diagrams at the standard temperature of 77 K for MnMoO_4_/rGO are also shown in Figure 3b. BET calculations show the specific surface area of about 78 m^2^/g and the average pore size is about 6.9 nm.

### 2.2. Electrochemical Studies

#### 2.2.1. Electrode Preparation

The three-electrode system used in this research included glassy carbon electrodes (GCE) with a diameter of 2 mm, an Ag/AgCl electrode, and a platinum wire electrode with a diameter of 1 mm as working, reference, and auxiliary electrodes, respectively. To modify the working electrode, 0.1 g of each of MnMoO_4_ and MnMoO_4_-rGO catalysts were dispersed in a specific volume of isopropyl alcohol and 15 µL Nafion solution (5%) and for 30 min by ultrasonication. A volume of 5 microliters of the resulting slurry was put on the surface of the GCE electrode and after drying in room temperature, electrochemical tests were performed.

#### 2.2.2. MnMoO_4_ and MnMoO_4_-rGO Nanocatalysts in MOR and EOR Processes

**Figure 4:**
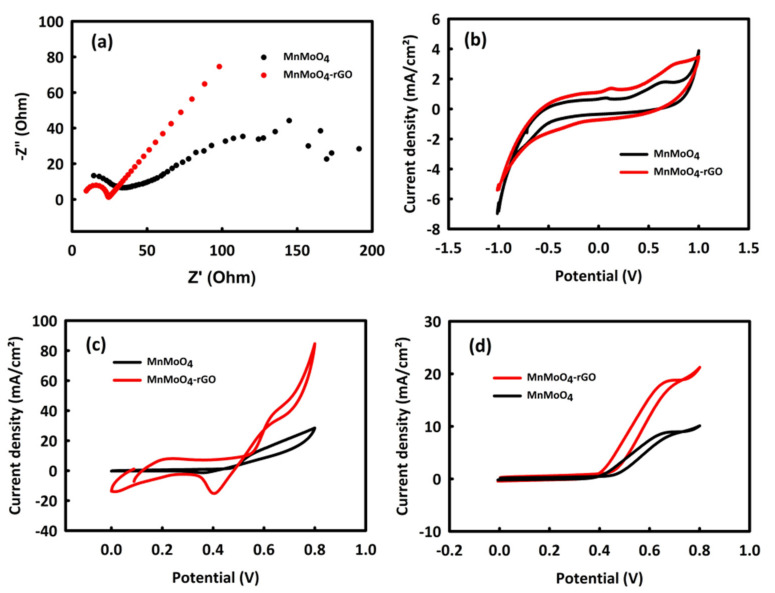
EIS (**a**) and CV (**b**) curves of MnMoO_4_ and MnMoO_4_-rGO in 0.5 M KOH; CV curves of MnMoO_4_ and MnMoO_4_-rGO in 0.5 M KOH/0.4 Methanol (**c**) and 0.5 M KOH/0.4 ethanol (**d**).

In general, catalysts based on metal oxides and without platinum have good activity in the oxidation of alcohols in an alkaline environment. The behavior of the electrodes modified with MnMoO_4_ and MnMoO_4_-rGO nanocatalysts was investigated in an alkaline medium 0.5 M KOH (potassium hydroxide) solution. Figure 4a,b show the behavior of these nanocatalysts in an alkaline environment investigated by electrochemical impedance spectroscopy (EIS) and cyclic voltammetry (CV). Nyquist plots formed from a quarterly circle in high frequency and a straight line in low frequency. The quarterly circle is related to charge transfer resistance and a straight line is related to Warburg impedance, indicating diffusion impedance. The values of charge transfer resistances (Rct) for MnMoO_4_ and MnMoO_4_-rGO are 26.52 and 15.74 Ω, respectively. In the CV analysis, it was observed that the capacitive and faradic current density for MnMoO_4_-rGO is higher than the current density of MnMoO_4_. Therefore, both analyses demonstrate the improvement of the catalyst performance by adding rGO, which facilitates the electrochemical processes by enhancing the electrochemically active surface area and increasing the electrical conductivity [37,38] in the MnMoO_4_-rGO catalyst.

In the next step and to prove the better efficiency of MnMoO_4_-rGO catalyst compared to MnMoO_4_ and before optimizing the concentration of methanol and ethanol, a solution containing 0.5 M KOH and 0.4 M of each alcohol was prepared. The CV behavior of MnMoO_4_-rGO and MnMoO_4_ nanocatalysts in the presence of methanol and ethanol at the scan rate of 20 mV/s is shown in Figure 4c,d. The oxidation peak of methanol and ethanol for MnMoO_4_-rGO has a much higher current density than MnMoO_4_. Therefore, in the subsequent tests, the electrochemical behavior of this nanocatalyst is more investigated in the process of methanol and ethanol oxidation.

**Figure 5:**
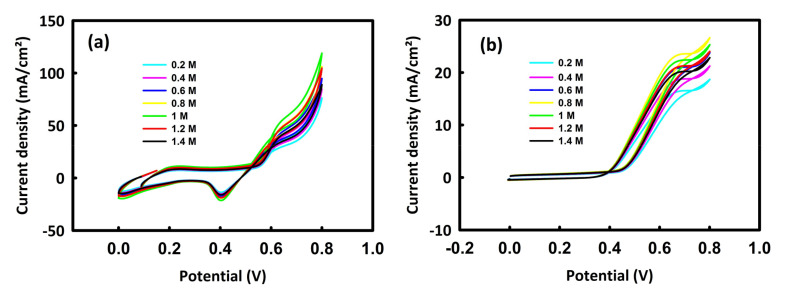
CV curves of MnMoO_4_-rGO in 0.5 M KOH/different concentrations of methanol (**a**) and 0.5 M KOH/different concentrations of ethanol (**b**).

First, solutions containing 0.5 M KOH and different concentrations of methanol or ethanol were prepared. Figure 5a relates to the cyclic voltammetry of the nanocatalyst in different concentrations of methanol. As can be seen, with the increase in methanol concentration from 0.2 M to 1 M, the trend of peak current density is upward, and from this concentration onwards, a decrease in current density is seen. Figure 5b also shows the behavior of the MnMoO_4_-rGO nanocatalyst in different concentrations of ethanol. In the oxidation of ethanol, it can be seen that the peak current density has an upward trend up to the concentration of 0.8 M ethanol, and from this critical concentration onwards, a decrease in the anodic current density is seen. At critical concentrations of methanol and ethanol, the surface of the nanocatalyst is likely saturated and the electron transfer process is disturbed [39]. Moreover, the alcohol’s oxidation by-products may occupy the porosities of the catalyst, which, in turn, reduces the oxidation current density. In this way, the concentrations of 1 M methanol and 0.8 M ethanol are selected as the optimal concentrations to investigate the oxidation of methanol and ethanol by MnMoO_4_-rGO.

**Figure 6:**
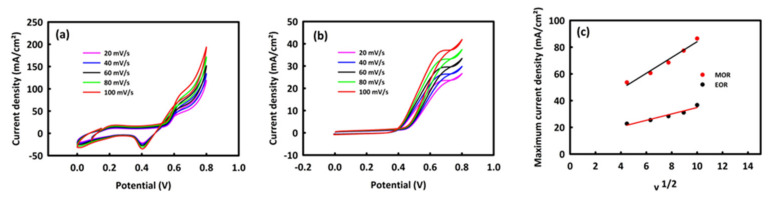
CV curves of MnMoO_4_-rGO in 0.5 M KOH at different scan rates at the presence of 1 M methanol (**a**) and 0.8 M ethanol (**b**) and current density versus square root of scan rate (**c**).

To study the electrocatalytic behavior of the catalyst for methanol and ethanol oxidation, CV analysis was performed at different scan rates in optimal concentrations of each of the alcohols. By increasing the scan rate, an increase in the anodic current density is seen in the process of methanol and ethanol oxidation (Figure 6a,b). The graph of the maximum current density according to the square root of the scan rate is shown in Figure 6c. There is a linear relationship between these two parameters with R^2^ = 0.972 and R^2^ = 0.958 for methanol and ethanol oxidation, respectively, indicating the diffusion-control mechanism in both processes.

In further explanation of the oxidation mechanism of alcohols on these catalysts, the following steps can be noted. In the first step, methanol and ethanol are adsorbed on the catalyst surface and in the porosity of the catalyst. In the next step, the adsorbed methanol and ethanol are deprotonated in the presence of hydroxyl ions and byproducts such as CH_3_O, CH_2_O, CHO, CH_3_CH_2_O, CH_3_CHO, CH_3_CO-, etc., create in this step. Moreover, hydroxyl ions are also adsorbed on the surface of the catalyst and form catalyst/OH-ads. In the third step, the surface of the catalyst is cleaned from adsorbed species.

**Figure 7:**
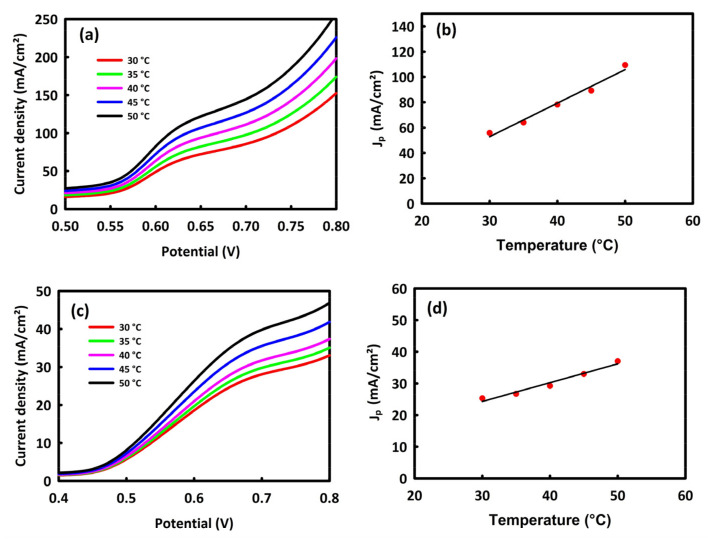
LSV analyses with a scan rate of 40 mV/s at different temperatures for the MOR process (**a**) and EOR process (**c**). The temperature in terms of the maximum current density for MOR process (**b**) and EOR process (**d**).

An increase in temperature is one of the parameters that facilitate the process of oxidation of alcohols and absorption of OH^−^ in an alkaline environment. To investigate the effect of temperature on MOR and EOR processes from room temperature to 50 °C, linear sweep voltammetry (LSV) analyses of the nanocatalysts were performed at a scan rate of 40 mV/s (Figure 7a,c). From these figures, an increase in the anodic current density is observed with increasing temperature, which indicates the kinetic of alcohol oxidation is facilitated by increasing temperature. The temperature and the peak current density (J_p_) have a linear relationship, which can be seen in Figure 7b,d.

**Figure 8:**
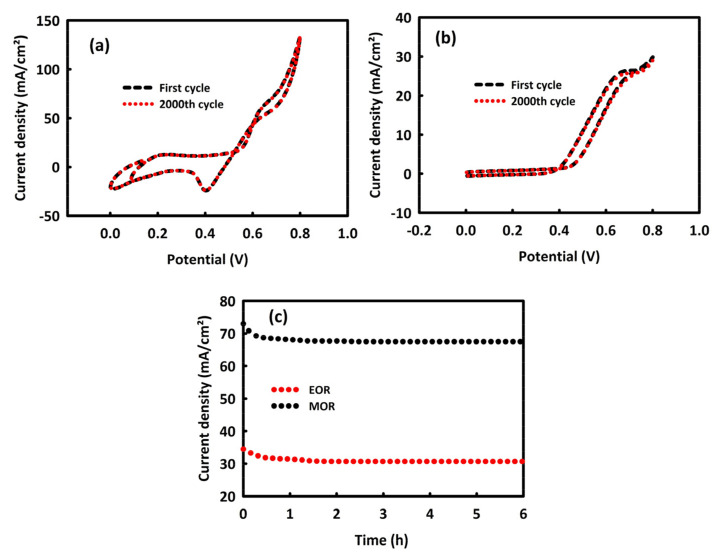
Two-thousand consecutive CV cycles in 0.5 M KOH/1 M methanol (**a**) and 0.5 M KOH/0.8 M ethanol (**b**) at a scan rate of 40 mV/s. chronoamperometry analysis in oxidation peak in 0.5 M KOH/1 M methanol and 0.5 M KOH/0.8 M ethanol (**c**).

The stability of the MnMoO_4_-rGO nanocatalyst in the MOR was evaluated by performing 2000 consecutive CV cycles in 0.5 M KOH/1 M methanol and at a scan rate of 40 mV/s (Figure 8a). MnMoO_4_-rGO nanocatalyst has a stability of 99.3% in MOR. This level of stability in the EOR process and at the concentration of 0.5 M KOH/0.8 M ethanol is equal to 97.4% (Figure 8b), which is an interesting value. The stability of MnMoO_4_-rGO nanocatalyst in MOR and EOR processes was also investigated by chronoamperometry analysis in a period of 6 h at the constant potential of 0.7 V, which is in agreement with the cyclic voltammetry results. The current density of catalyst for MOR and EOR steadily decreases, likely as a result of hydrogen adsorption and double-layer discharge. The current density for MOR at a steady state is higher than that of EOR, which supports the result of cyclic voltammetry. MnMoO_4_-rGO has 91.7% stability in the MOR and 88.6% in EOR (Figure 8c), which are acceptable values. This good stability is assigned to the incorporation of rGO in MnMoO_4_, likely increasing the stability of electrocatalytic activity for MOR and EOR.

The efficiency of MnMoO_4_-rGO nanocatalyst in MOR and EOR processes have been compared with other similar studies in Table 1. It is to be noted that the values of current density and peak potentials are comparable with other reported works.

## 3. Experimental

### 3.1. Materials and Instruments

Sodium molybdate dihydrate (Na_2_MoO_4_•2H_2_O), manganese (II) nitrate hexahydrate (Mn(NO_3_)_2_•6H_2_O), urea (CH_4_N_2_O), and potassium hydroxide (KOH) with a purity of more than 99% were obtained from Sigma Aldrich (Massachusetts, United States). Methanol and ethanol alcohols were also obtained from Merck (Darmstadt, Germany). The crystal structure was evaluated by X-RAY diffraction (XRD) (PANalytical X’Pert Pro, Almelo, The Netherlands) and the surface morphology of nanocatalysts was determined by scanning electron microscopy (SEM-TESCAN, Brno, Czech Republic). Transmission electron microscopy (TEM) were performed by Philips EM 208S (Eindhoven, The Netherland). Electrochemical analyzes were performed by potentiostat/galvanostat Autolab 302 N (Metrohm, Herisau, Switzerland) with a three-electrode system.

### 3.2. Synthesis of MnMoO_4_ and MnMoO_4_-rGO Nanocatalysts

To synthesize the MnMoO_4_ nanocatalyst by hydrothermal method, 0.4 g of sodium molybdate, 0.4 g of manganese nitrate, and 1 g of urea were dissolved in 40 mL of deionized water for 30 min. The resulting solution in an 80 mL autoclave reactor and put in the oven at a temperature of 150 °C for 12 h. MnMoO_4_-rGO was also synthesized in the same way, with the difference being that at first, 0.03 g of graphene oxide was added to the precursor’s solution. After cooling down the reactor at ambient temperature, the products were washed three times with water and ethanol by centrifuge and decantation to remove unreacted precursors. Then, it was dried at 60 °C for 10 h. The resulting powders were calcined for 2 h at 300 °C. It is worth mentioning that graphene oxide was synthesized by a modified Hummers’ method. An amount of 1 g of graphite powder was dispersed in a mixture of sulfuric acid and phosphoric acid under magnetic stirring for 1 h. The vessel was put in an ice bath and 9 g of KMnO_4_ was slowly added to it. The mixture was stirred for 24 h and after that 100 mL of deionized water was slowly added to it. Then, to eliminate the excess KMnO_4_, 35 mL of hydrogen peroxide was added and stirred for 10 min. The mixture was centrifuged and the supernatant was decanted. The residual was washed with 0.2 M hydrochloric acid and water and dried in the oven at 90 °C.

## 4. Conclusions

The use of metal oxides as catalysts in the field of energy and especially as electrodes in the structure of supercapacitors and as catalysts in the anode and cathode of methanol and ethanol fuel cells is one of the most important topics of our research team. The result of this research is the proposal of a cheap nanocatalyst based on metal oxide and reduced graphene oxide for use in the anode of alcohol fuel cells. Our proposed catalyst is MnMoO_4_-rGO, which is synthesized by the hydrothermal method. It showed the oxidation of methanol at a peak potential of 0.62 V with a current density of 60.59 mA/cm^2^ and the oxidation of ethanol at a peak potential of 0.67 V and a current density of 25.39 mA/cm^2^ at the scan rate of 40 mV/s. The values of charge transfer resistances (Rct) for MnMoO_4_ and MnMoO_4_-rGO obtain 26.52 and 15.74 Ω, respectively. MnMoO_4_-rGO exhibited very good stability in chronoamperometric analysis during 6 h with 91.7% stability in MOR and 88.6% in EOR. The electrocatalytic activity of the MnMoO_4_ was improved by adding rGO to its structure. By increasing the active surface area and improving electrical conductivity, rGO facilitates the oxidation of methanol and ethanol. These types of materials can be promising and attractive catalysts for more investigations in alcohol fuel cells. The use of catalysts similar to MnMoO_4_-rGO in the oxidation of other fuels used in fuel cells, such as the oxidation process of urea, glucose, glycerol, etc., can open an attractive path for researchers in the field of catalysts and energy.

## Figures and Tables

**Table 1:** Comparison of MOR and EOR performances of MnMoO_4_-rGO nanocatalyst with other similar works.

Electrocatalyst	Electrolyte Composition	Peak Potential (V)	Current Density (mA cm^−2^)	Scan Rate (mV/s)	Reference
MnMoO_4_-rGO	1 M Methanol/0.5 M KOH	0.62	60.59	40	This work
MnMoO_4_-rGO	0.8 M Ethanol/0.5 M KOH	0.67	25.39	40	This work
Mn_3_O_4_-CeO_2_-rGO	0.8 M Methanol/1 M KOH	0.51	17.7	90	[40]
rGO-NiO/CuO MOF	3 M Methanol/1 M NaOH	0.9	437.28	50	[41]
Mn_3_O_4_-Co_3_O_4_-rGO	1 M Methanol/0.5 M KOH	0.48	16.5	100	[42]
α-CoMoO_4_ nanoflakes	1 M Methanol/0.5 M KOH	0.8	25	50	[43]
NiO-CuO	0.3 M Methanol/0.5 M NaOH	0.6	12.2	50	[44]
MnCo_2_O_4_/NiCo_2_O_4_/rGO	2 M Methanol/2 M KOH	0.58	24.76	20	[45]
Pd-Ni Fe/MnO_2_/Vulcan	0.2 M KOH/1 M Ethanol	+0.05–+0.3	3.03	50	[46]
Ni_3_S_4_-NiS-rGO	0.7 M Methanol/1 M KOH	0.54	55	60	[47]
NiMoO_4_	1 M KOH/2 M Methanol	0.45	49	50	[48]
Ni_6_MnO_8_	1 M KOH/1 M Ethanol	1.53	13.69	50	[49]

## Data Availability

Data are available on request from the authors.
